# Micropenis: Etiology, Diagnosis and Treatment Approaches

**DOI:** 10.4274/Jcrpe.1135

**Published:** 2013-12-12

**Authors:** Nihal Hatipoğlu, Selim Kurtoğlu

**Affiliations:** 1 Erciyes University Faculty of Medicine, Department of Pediatric Endocrinology, Kayseri, Turkey

**Keywords:** Micropenis, etiology, diagnosis, treatment

## Abstract

Micropenis is a medical diagnosis based on correct measurement of length. If stretched penile length is below the value corresponding to - 2.5 standard deviation of the mean in a patient with normal internal and external male genitalia, a diagnosis of micropenis is considered. Micropenis can be caused by a variety of factors including structural or hormonal defects of the hypothalamic-pituitary-gonadal axis. It can also be a component of a number of congenital syndromes. For the etiological evaluation, endocrinologic tests are important. This article reviews the etiology, diagnosis, treatment and management of micropenis.

**Conflict of interest:**None declared.

## INTRODUCTION

Micropenis is a medical diagnosis often incorrectly made. A misdiagnosis may cause parental anxiety and may lead to unnecessary examinations and tests. The correct diagnosis is made by measuring stretched penile length. The first description of standard penile length for age was used by Schonfeld and Beebe (1). In time, the definition of micropenis was accepted as a penile length smaller than 2.5 standard deviations (SD) below the mean (2). Micropenis may occur as an independent abnormality by itself or as a clinical finding of many syndromes. In the Unites Stated of America (USA), the incidence of micropenis was reported as 1.5 in 10 000 male children born between 1997 and 2000 (3). During embryonic development, following the differentiation of bipotential gonadal ridge to testis, placental human chorionic gonadotropin (hCG)-driven testosterone synthesis begins in Leydig cells at 8-12 weeks, resulting in penile differentiation stimulated by dihydrotestosterone (DHT), a product of the transformation. Fetal androgen levels are high between the 8th and 24th weeks of gestation, with peak levels often observed between the 14th and 16th weeks. Consequently, there is a marked increase in penile length during the second and third trimesters, with an increase of approximately 20 mm from weeks 16 to 38 (4,5). It can thus be deduced that a true micropenis is caused by a hormonal abnormality that occurs after the 12th week of gestation (6).

Hormonal activity of the hypothalamic-pituitary axis and that of the testes increases within the first 6 months of postnatal life (7). The reason for the activation of the axis is, due to pituitary gonadotropin secretion, cessation of the negative feedback effects of both the placental sex steroids and peptides. An increase in both testis volume and penile length is observed physiologically during this active phase (7). During this period, follicle stimulating hormone (FSH) and luteinizing hormone (LH) levels rise increasing the circulating testosterone, inhibin B, and anti-Mullerian hormone (AMH) levels, sometimes even to higher levels than in adult males (8,9). Testosterone levels increase in parallel to the activation peak between the 1st and 3rd months and decrease to prepubertal levels from the 4th-6th months onwards (10).

**Criteria for Diagnosis**

**Figure 1:**
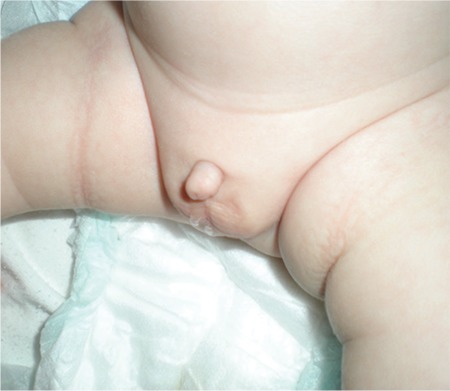
Micropenis in a newborn (from the archives of the Department of Pediatric Endocrinology, Erciyes University, Faculty of Medicine)

Early diagnosis of “true micropenis” is important, because it allows for various treatment options to be utilized early. The first step in the diagnosis of micropenis is the physical examination of the patient’s external genitalia. Micropenis refers to a condition which occurs only in XY males. It is characterized by a small penis and a median raphe, foreskin, as well as normal localization of the urethral meatus opening (Figure 1) (11).

Micropenis may have a retracted or flaccid appearance, depending on the length of the shaft and its being erect or non-erect. Presence or absence of corpus cavernosa and corpus spongiosum may also affect the appearance of the penis. The scrotum is present and fused normally, but it may be underdeveloped (hypoplastic). Typically, the testicles are in the scrotum, but they may or may not function normally. However, in some patients, testicular descent may be impaired due to a syndromic condition or to severe hormonal effects (12). Testicular volume is also expected to be below normal measurements. There often is no evidence of feminization (13,14).

**Measurement of Penile Length and Values Reported in Healthy Infants and Children**

Correct measurement of penile length is important because the diagnosis of true micropenis depends on it. A correct and ccurately measured penile length of -2.5 SD below the mean for age and presence of internal and external genital organs compatible with a 46,XY karyotype are sufficient findings to support a diagnosis of micropenis (12).

**Figure 2:**
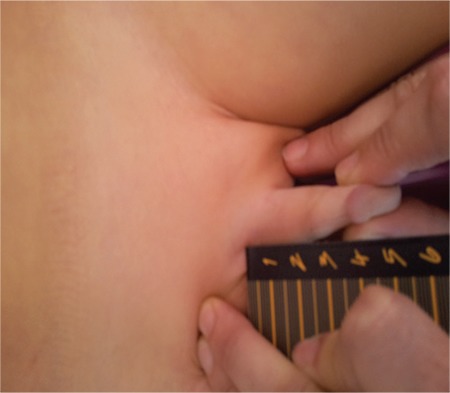
Correct technique for measuring penile length (from the archives of the Department of Pediatric Endocrinology, Erciyes University, Faculty of Medicine)

**Figure 3:**
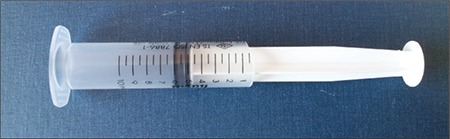
A modified syringe to be used for measuring penile length

Traditional methods utilize a ruler or caliper to measure penile length. Penile length should be measured when the penis is fully stretched, not flaccid; the glans penis should be held with the thumb and forefinger, and the measurement should be taken from the pubic ramus to the distal tip of the glans penis over the dorsal side. The suprapubic fat pad should be pressed inwards as much as possible, and if present, the foreskin must be retracted during the measurement (Figure 2) (12,15). While penile diameter and its ratio to length are typically normal, the diameter may rarely be smaller in cases with severe hypoplasia of the corpus cavernosum (14). A different approach involves the use of a 10 mL disposable syringe. The needle-side tip of the syringe is cut off, and the piston is inserted into the syringe on the cut side (Figure 3). The open side of the syringe is placed on the penis. The piston is pulled back while pressing the fat pads inwards, which causes the penis to be pulled inside the syringe as a result of suction. Once the penis is stretched inside the syringe, penile length is read from the scale added on the modified syringe. This technique allows for the elimination of measurement differences caused by the suprapubic fat pad (16).

The obtained value for penile length is compared with the normal values for the chronological age group. Penile length must be -2.5 SD below normal for the penis to be accepted as a micropenis. Table 1 shows the normal values by age and minimum and maximum lengths corresponding to -2.5 SD (17). In addition, mean penile lengths and percentiles in adolescents are presented in Table 2 as reported by Lee and Reitor (18). In a recent study by Kutlu (19) investigating normal penis lengths in 514 Turkish newborns, measurements were taken within the first 24 hours after birth, and the results showed that the mean±SD value was 3.77±0.35 cm. The penile length corresponding to the -2.5 SD value was 2.9 cm. In a second study on 1217 Turkish healthy term newborns (20), the mean stretched penile length was reported as 3.16±0.39 cm, the value corresponding to -2.5 SD was 2.19, and the value corresponding to +2.5 SD was 4.14 cm. The results of a study on 1278 Turkish healthy prepubertal children (21) are given in Table 3.

**Differential Diagnosis**

Loose penile skin that does not stretch tightly around the body of the penis, penile skin being insufficient or imperfect, excessive fatty tissue, formation of scar tissue following a penile surgery, and presence of a web of skin underneath the penis are conditions also referred to as “inconspicuous penis”, and these conditions must be differentiated from micropenis (22,23).

**Figure 4:**
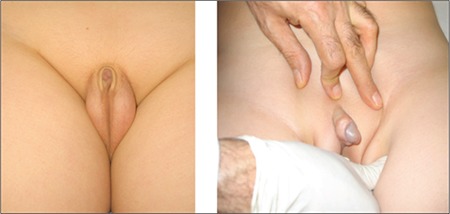
Buried penis in an obese child (from the archives of the Department of Pediatric Endocrinology, Erciyes University, Faculty of Medicine)

Children who present with a suspicion of micropenis are often prepubertal and obese, and the small size of their penis is caused by the pressure of the prepubic fat on the penis. Correct measurement of penile length and careful physical evaluation may help differentiate this condition from micropenis. In this condition, also known as “buried penis”, true penile structure can be revealed by pressing the surrounding fatty tissue inwards as much as possible (Figure 4) (22).

**Figure 5:**
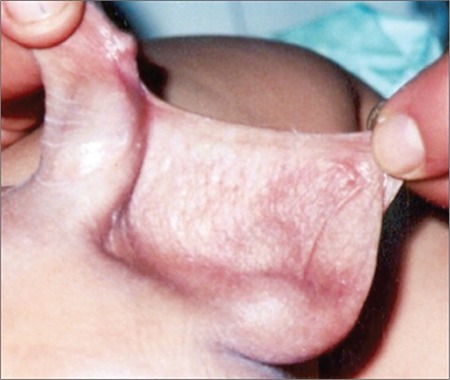
Webbed penis (from reference 23)

Suprapubic fat pads surrounding the penis in the absence of additional skin for the shaft is referred to as “trapped penis”. It is a condition where the body of the penis is entrapped within the scarred prepubic skin following circumcision or a trauma. It is the result of excessive surrounding due to the cohesion between the scrotal and penile skin. Another condition that must be considered in the differential diagnosis is the “webbed penis”, characterized by a skin tissue connecting the penis to the front side of the scrotum (Figure 5) (23,24).

Penile agenesis, or absence of the penis and curvature of the head of the penis, or chordee, are rare conditions which should also be considered in the differential diagnosis (24).

**Etiology**

Causes of true micropenis can be examined under three headings: hypogonadotropic hypogonadism due to pituitary/hypothalamic insufficiency, hypergonadotropic hypogonadism due to primary testicular insufficiency, and idiopathic (Table 4) (12,14,15,25).

**Diagnostic Tests**

**Laboratory Tests**

First-line tests include measurement of serum gonadotropins, testosterone, DHT, and precursors of testosterone. Levels of other pituitary hormones may also be measured when needed.

Endocrinologic assessment helps determine at what level the cause of micropenis is in the hypothalamic-pituitary-testicular axis (9). In addition to evaluation of central endocrine functions, testicular functions also need to be evaluated simultaneously. Hence, serum testosterone levels are measured before or after administering hCG. This test is performed by intramuscular administration of hCG in a dose of 1 000 units for 3 days, or 1 500 units every two days for 14 days; testosterone levels below 300 ng/dL may indicate gonadal dysgenesis (26). If LH and FSH levels are elevated, and there is no increase in testosterone levels following administration, testicular insufficiency or absence should be considered. In addition, measuring 17 hydroxyprogesterone, dehydroepiandrosterone, and androstenedione levels before or after a hCG stimulation test can reveal enzyme defects that play a role in testosterone synthesis.

Inhibin B and AMH, also known as Mullerian-inhibiting hormone are produced by functional Sertoli cells, and determination of their blood levels can be used to detect the presence of functional testicular tissue (9,27). Low levels of AMH, accompanied by normal inhibin B levels, and a rare defect in the AMH gene, indicate persistent Mullerian duct syndrome (9).

**Imaging Tests**

Pelvic ultrasound can be used to visualize internal genital organs in suspicious cases. Magnetic resonance imaging is used to investigate structural midline defects, such as pituitary stalk dysplasia syndrome, central diabetes insipidus characterized by absence of the pituitary bright spot in the posterior neurohypophysis, and pituitary dysplasia (9, 28). A small posterior pituitary gland, thinned or disappeared pituitary stalk, and posterior pituitary ectopia are findings that may indicate hypopituitarism, thus enabling determination of the etiology (28,29).

**Genetic Tests**

Some authors suggest karyotype assignment using chromosomal analysis or Y-fluorescence in order to determine the sex. Genetic testing may be necessary to eliminate other syndromes (24).

**Treatment Approaches**

The goals of treatment for micropenis are to provide a body image that will not cause embarrassment for the patient when seen by others, to enable the patient to have normal sexual function, and also enable the patient to urinate standing up. Not exactly reaching the mean penile length of the healthy population does not mean failure.

**Testosterone Treatment**

Presence or absence of a response to androgens during infancy is crucial for sex assignment (30). Testosterone is initially administered for a short period of time in order to evaluate the response of the penis. Administration can be by intramuscular injection or topical application. In order to observe initial progress, four doses of 25 mg of testosterone cypionate or enanthate in oil are administered intramuscularly once every 3 weeks for 3 months. Side effects are minimal; however, it may cause temporary acceleration in growth rate and in advancement of bone age (31). Sultan et al (26) state that there was no consensus on the dose, method of administration, or duration in testosterone therapy for micropenis. Guthrie et al (31) have suggested a treatment scheme of 25 mg of intramuscular depot-testosterone administered every three weeks for three months. Main et al (32) reported successfully achieving increases in both penile length and scrotum width in three boys who were diagnosed with hypogonadotropic hypogonadism [congenital hypogonadotropic hypogonadism (CHH) and panhypopituitarism] and had no penile growth or scrotal fold formation after birth, by administering 1-5 mg of testosterone suppository daily. However, these authors stated that they observed no changes in testicular volume nor in Sertolian hormone (inhibin B and AMH) levels.

Typically, a good response is a 100% increase in penile length in the course of the initial treatment (32,33). Another author (26) considers a 3.5-cm increase in penile length by testosterone injections as an adequate response. In case of an inadequate response, applications may be repeated within a short period of time (9,33).

Topical testosterone application is effective during infancy. Arisaka et al (34) demonstrated increases in penile lengths in 50 infants and children aged between 5 months and 8 years, by administering 5% testosterone cream for a duration of 30 days. Testosterone that is absorbed transdermally was shown to stimulate growth hormone (GH) secretion from the pituitary gland and promote bone growth by increasing insulin-like growth factor-1 production. Therefore, it can be said that long-term dermal application of testosterone promotes skeletal growth, as well as penile growth (34).

Clinical studies have, so far, shown that testosterone treatment had positive effects on penile growth during infancy. However, these studies do not show whether this growth continues during adolescence and adulthood (35). Androgen resistance or androgen receptor defects are likely in cases that show no response, and there is a possibility of insufficient virilization during puberty in such cases. An important aspect of testosterone treatment is the initiation of the treatment in early infancy and childhood. Penile androgen expression decreases in patients with hypogonadotropic hypogonadism. There is a natural decrease in androgen receptors during the early adulthood period, and early application of testosterone allows for penile androgen receptor concentration to increase. Therefore, treatment before this period of decrease is recommended (22).

**Topical 5-a Dihydrotestosterone (DHT) Gel**

In prepubertal patients with androgen insensitivity, topical application of DHT gel to the periscrotal region 3 times daily for a total of 5 weeks has been shown to increase serum DHT levels. In one study (36), an increase in penile length and an acceleration in genital development in a male infant with 16 XY karyotype was reported following the treatment regime mentioned above. This treatment regime was also demonstrated to be effective in patients with 5-alpha-reductase deficiency (5-αRD). Bertelloni et al (37) demonstrated that the DHT cream achieved an increase of at least 120% in penile length in three Italian newborns with 46,XY karyotypes, two of whom had 5-αRD. In another study, percutaneous 2.5% DHT gel was used in 6 children, aged between 1.9 and 8.3 years, with micropenis of different etiologies. An increase in phallic growth was observed when one daily dose of 0.2-0.3 mg/kg DHT was used for 3-4 months (37). Side effects were reported to be similar to that of testosterone treatment, except for minimal effects such as minor skin irritations (38). This treatment option might be a good alternative for patients who do not respond to testosterone.

**LH-FSH Applications**

Recombinant human FSH-LH treatment during the first few years of life promotes an increase in testicular growth and penile length in patients with hypogonadotropic hypogonadism, although this effect is not very significant.

Main et al (27) reported an increase in penile length from 1.6 cm to 2.4 cm and a 170% increase in testicular volume evaluated by ultrasonography in a patient with micropenis, when testosterone treatment was added to subcutaneous injections of 20 and 21.3 IU of recombinant LH and FSH twice a week, for a duration of 6 months. The authors also demonstrated increases in LH, FSH, and inhibin B levels. The treatment was tolerated well, even though certain side effects, such as increased amount of body hair, increased pigmentation, and intermittent vomiting, were noted.

Although exogenous hormone therapy in patients with micropenis increases penile growth, the length of the penis may still be below the mean length of the normal adult population (36).

Bougneres et al (39) planned a treatment design aiming to physiologically achieve peak postnatal gonadotropin levels, also referred to as mini-puberty. The study included two cases - one with congenital hypopituitarism and the other with isolated CHH, both diagnosis being based on findings of micropenis and cryptorchidism. The patients were administered recombinant human LH and FSH, subcutaneously, by a pump for 6 months starting from the newborn period. Testicular volumes of 0.45 and 0.57 mL at birth increased to 2.1 mL in 7 months, and penile length of one of the patients increased from 8 mm to 30 mm, and the other patient’s penile length increased from 12 mm to 48 mm. While mean serum LH and FSH levels were normal and supranormal, respectively, the mean testosterone levels increased from undetectable to normal levels, and inhibin B and AMH levels increased up to normal levels for the age group (40).

**Surgical Treatment**

If the micropenis does not reach an adequate length despite medical interventions, surgical treatment options are considered. The first reconstructive surgery was reported by Hinman (41) in the early 1970s when he performed reconstruction on a patient with micropenis. In the 1980s, in the surgical area of penile reconstruction, a technique was developed where a new fasciocutaneous phallus was reconstructed using the radial artery forearm flap (42). Despite other techniques involving different flaps such as the sensate osteocutaneous fibula, scapular free, suprapubic abdominal wall, and vertical rectus abdominis, the radial artery forearm flap has remained the most popular of all (43).

Cosmetic and functional outcomes reached acceptable levels particularly when an implant was used following reconstruction with prosthesis (44). Even though the technique is utilized in select patients, the chance of a complication is high even in the hands of an experienced surgeon (44,45).

In general, patients remain displeased with the appearance of their genitals (46). However, current evidence indicates that, even if micropenis has remained as such, the majority of patients who are raised as males have normal sexual identities and functioning.

In summary, micropenis is a medical diagnosis which is dependent on correct measurement. It may be an independent abnormality or a part of many syndromes. Micropenis can occur as a result of pituitary/hypothalamic insufficiency, primary testicular insufficiency, or can be idiopathic. Endocrinologic assessment helps in determining the etiology of micropenis. Early diagnosis is important for various treatment options. 

## Figures and Tables

**Table 1:**
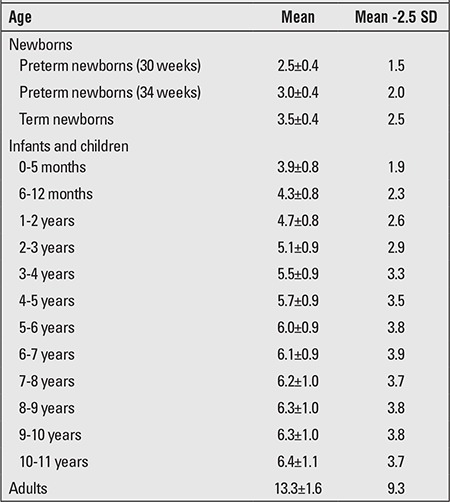
Mean and calculated -2.5 standard deviation (SD) values for stretched penile length (cm) (17)

**Table 2:**
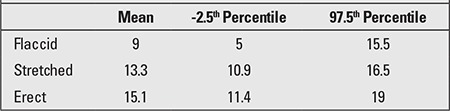
Flaccid, stretched, and erect penile lengths (cm) in healthy adolescents (18)

**Table 3:**
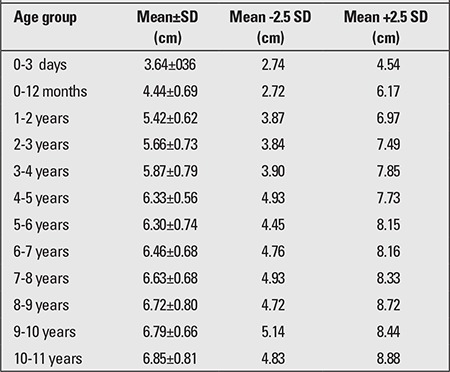
Penile measurements in prepubertal Turkish children (21)

**Table 4:**
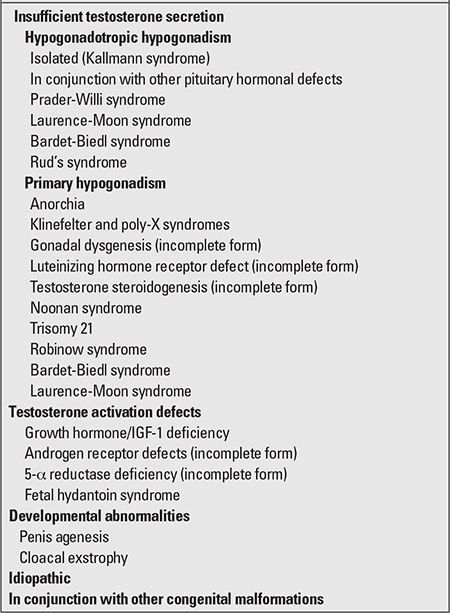
Causes of micropenis (15)
